# Eye tracking in early autism research

**DOI:** 10.1186/1866-1955-5-28

**Published:** 2013-09-26

**Authors:** Terje Falck-Ytter, Sven Bölte, Gustaf Gredebäck

**Affiliations:** 1 Department of Women’s & Children’s Health, Center of Neurodevelopmental Disorders at Karolinska Institute (KIND), Pediatric Neuropsychiatry Unit, Child and Adolescent Psychiatry Research Center, Gävlegatan 22, Stockholm, SE-11330, Sweden; 2 Uppsala Child & Babylab, Department of Psychology, Uppsala University, Uppsala, Sweden; 3 Division of Child and Adolescent Psychiatry, Stockholm County Council, Stockholm, Sweden

## Abstract

Eye tracking has the potential to characterize autism at a unique intermediate level, with links ‘down’ to underlying neurocognitive networks, as well as ‘up’ to everyday function and dysfunction. Because it is non-invasive and does not require advanced motor responses or language, eye tracking is particularly important for the study of young children and infants. In this article, we review eye tracking studies of young children with autism spectrum disorder (ASD) and children at risk for ASD. Reduced looking time at people and faces, as well as problems with disengagement of attention, appear to be among the earliest signs of ASD, emerging during the first year of life. In toddlers with ASD, altered looking patterns across facial parts such as the eyes and mouth have been found, together with limited orienting to biological motion. We provide a detailed discussion of these and other key findings and highlight methodological opportunities and challenges for eye tracking research of young children with ASD. We conclude that eye tracking can reveal important features of the complex picture of autism.

## Review

### Introduction

Autism spectrum disorder (ASD) is a neurodevelopmental condition defined by impairments across the areas of reciprocal social interaction and verbal and non-verbal communication, alongside repetitive and stereotyped behaviors [1]. Intervention, particularly early intervention, may improve long-term outcomes for individuals with ASD [2]. Early identification is obviously a prerequisite for early delivery of intervention, which has led to the recent focus on infant development and detection of ASD in infancy and early childhood. Different types of research drive knowledge acquisition about early autism. One approach is to study very young children with ASD diagnoses while another is based on retrospective analyses, including analyses of home videos. A third approach is to longitudinally follow infant siblings of children with ASD, who are at increased risk for ASD [3,4]. This last approach has substantially advanced our knowledge of developmental trajectories in children at risk for ASD during the first years of life (for reviews, see [5-7]). In brief, we now know that during the first year of life, ASD is associated with altered (neuro-)developmental trajectories in diverse domains including motor, language, cognitive and socio-communicative functions [5,8-12]. During the same period, evidence exists for structural brain differences between infants who are later diagnosed with ASD and those who are not [13].

Understanding how infants and children use their eyes in various contexts is important to understanding their opportunities for learning and development [14-16]. An effective way to study looking performance is to use eye tracking technology. Eye tracking allows researchers to measure how the observer distributes gaze and can serve to address a wide range of scientific questions [17-19]. Recently, several eye tracking studies of young children with ASD have been published, illustrating an emerging consensus that detailed characterization of young children with ASD at the level of eye movements is important.

Corneal reflection eye tracking is the most common method used to study gaze performance in infants and young children [20,21]. This method estimates the location of gaze with high accuracy (precision <1 visual degree, sampling rate 50 to 300 Hz) based on the reflection of near-infrared light from the cornea and the pupil. Gaze position is calculated by computer algorithms based on video recordings (showing the pupil and the near-infrared light reflections) collected by remote cameras placed in front of the observer. Thus, there is no need for head-mounted equipment or other obtrusive devices that reduce the comfort of infants and children and their willingness to participate. The use of corneal reflection eye tracking is not new [22,23], but recent advances in computer capacities and eye tracking algorithms have promoted the development of several easy-to-use and robust eye tracking systems (for reviews, see [20,24]). Eye tracking both improves measures obtainable with less advanced methods (for example, coding from video) and adds measures not available by other means, including fine-grained scanpath and fixation analyses [25].

In this review, we critically assess the use of eye tracking in research focused on autism early in life. Eye tracking studies were identified through searches (through August 2013) in PubMed, Web of Science, and Google Scholar using ‘autism’ , ‘child’ , and ‘eye tracking’ as keywords [see Additional file 1 for a list of identified studies]. We devote the most attention to the studies that have contributed to significant knowledge advancement in a particular domain, that are part of a current debate or that effectively convey the opportunities or challenges with eye tracking research in this population. We do not address general methodological issues, which are covered extensively elsewhere, for example, [25].

Frequently, terms such as ‘looking’ , ‘gazing’ , ‘fixating’ , ‘eye movements’ and ‘attention’ are used more or less interchangeably. As a simplification, eye movements can be divided into fixations (stabilized gaze on static target), saccades (rapid eye rotation from one fixation to another) and smooth pursuit (stabilized gaze on moving target). Attention is a multifaceted construct that is not directly observable, but it is generally accepted that some eye tracking tasks capture specific aspects of visual attention [25,26].

We start with reviewing studies that employed (semi-) naturalistic stimuli. In these studies, the participants’ gaze is measured while they look at relatively complex scenes that resemble real life. These stimuli are typically not interrupted by experimental trials or conditions. In this section, we also review studies that have included static and dynamic faces shown in isolation. We then move on to studies that have used the paired visual preference paradigm, which can point to specific factors that influence viewing. All of these paradigms have in common that they, at least traditionally, use aggregated looking time in various areas of interest as the key outcome variable. This feature contrasts with event-related designs, in which properties of gaze shifts are the critical outcome variables, and we review this set of studies last.

**Figure 1:**
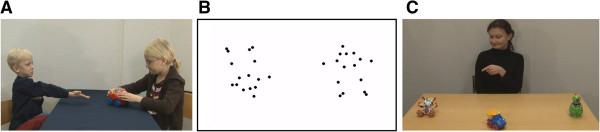
**Examples of stimuli used in eye tracking studies of young children with ASD. A)** Stimulus used to study semi-naturalistic scene viewing, in this case during observation of other children’s interactions. **B)** Stimulus used to study visual preference, in this case for biological motion and audiovisual synchrony. **C)** Stimulus used to study gaze/point following, key components of joint attention. Reproduced with permission from ref. [53], refs. [74,110], and ref. [90]. ASD, autism spectrum disorder.

Following each section, we discuss the general methodological and conceptual issues that arise, as well as future directions, and address more specific points in connection with the summary of the relevant study. Figure 1 shows examples of stimuli used in the three different types of studies.

### Studies of looking performance in (semi-) naturalistic contexts

In a seminal eye tracking study, Klin *et al*. [27] showed that the way high-functioning adults with ASD look at dynamic social scenes is markedly different from how typical adults look at the same scenes. This report has greatly influenced the field, and several of the studies reviewed in this section focus on how young children with ASD look at longer video clips with social content. All of the studies reviewed here have compared groups in terms of where they look on the stimuli, with much less attention to *when* they look there (a distinction we return to later). All studies have investigated social stimuli, including people and faces. The studies differ in whether they are exploratory or hypothesis driven and whether they involve efforts to systematically vary the information available in the stimuli.

Aiming to quantify attention to eyes of others in different young child groups, Jones *et al*. [28] compared children with ASD (n = 15; mean age 2.3 years), typical development (n = 24, mean age 2.1 years) and developmental delays (n = 15, mean age 2.1 years). The participants were shown videos with a total duration of approximately four minutes of a woman looking into the camera and speaking as if she were addressing the observing child, engaging in childhood games like ‘pat-a-cake.’ Around her were pictures and shelves of toys. The ASD group was matched to the developmentally delayed group both in terms of chronological age and verbal mental age and to the typically developing group on chronological age and non-verbal mental age. Compared to both other groups, the ASD group looked less at the woman’s eyes and more at her mouth (the latter was only marginally significant for the ASD–developmentally delayed comparison). In ASD, preference for eyes was associated with having less impairment according to the Autism Diagnostic Observation Schedule (ADOS; social interaction total score). Jones *et al*. suggested that the increased looking time at the mouth in the ASD group could be driven by preference for audiovisual synchrony. This hypothesis relates to another study by the same group, discussed below [29].

Taking a more explorative approach, Hosozawa *et al*. [30] compared children with ASD (n = 25, mean age 4.9 years), specific language disorder (SLL; n = 16, mean age 2.5 years), and typically developing children (n = 25, mean age 3.1 years) in terms of how they looked at people and faces embedded in several short video clips (six seconds each) excerpted from a film or TV program for children. The clips contained one to several people engaged in social interaction, such as conversation or speaking to the audience. Multidimensional scaling revealed a more heterogeneous looking pattern in ASD participants than in the other groups. Follow-up analyses indicated that children with ASD looked away from actors prematurely during speech episodes and looked less at faces in general compared to the other two groups. In contrast to the Jones *et al*. study [28], Hosozawa *et al*. did not find diminished eye looking or increased mouth looking in ASD. They found that the SLL group was characterized by more mouth looking and less eye looking than the other groups. The authors speculated that this outcome might reflect a way to compensate for limited speech processing skills in the children with SLL.

Chawarska *et al*. [31] provided an example of a semi-naturalistic eye tracking study that included systematic variations in the stimulus content. The goal was to study the effect of context on looking performance in children with ASD (n = 54, mean age = 1.8 years), typical development (n = 48, mean age = 1.7 years) and developmental delays (n = 20, mean age = 1.7 years). The ASD and developmentally delayed groups were matched on verbal and non-verbal mental age. The stimulus was a three-minute video of a woman seated behind a table, performing various actions. Around this actress were four shelves of toys. The authors split the analysis of looking time into episodes defined by the content of the video, forming four segment types. In the dyadic bid segments, the actor looked into the camera and spoke as if she were addressing the observing child. In the sandwich segments, she was making a sandwich. In the joint attention segments, she briefly looked at the camera before she moved her gaze to an object on one of the shelves, exclaiming ‘uh-oh’. Finally, in the moving toys segments, the actor made a gaze shift from the camera to the shelf opposite a moving toy. Compared to the other groups, children with ASD looked less at the actor’s face (and her mouth in particular). This effect was most clear in the dynamic bid condition, somewhat weaker in the joint attention condition and not present in the two remaining conditions. The authors argued that the degree to which communicative cues such as eye contact and speech were present could explain the difference across conditions. The ASD group looked more at the hand/object area than the other groups, in contrast with an eye tracking study by Shic *et al*. [32], which found less looking time at the hand/object area in ASD. Finally, Chawarska *et al*. [31] found that less looking time at the actor’s face and mouth was associated with having better expressive single-word vocabulary than receptive language capacity, a language profile that is typical for children with ASD in this age range [33].

Using the same stimuli and paradigm as in their previous study [33], Chawarska *et al*. [34] asked whether altered social looking performance in infancy was predictive of a later ASD diagnosis. They followed infants at elevated risk for ASD (siblings of children with ASD) as well as infants with low risk for ASD from 6- to 24-months of age. At the final point, a clinical best estimate diagnosis was made based on information about the ASD-related symptomatology and general developmental level. At six months, eye tracking was used to assess looking patterns during observation of videos with social content. Looking patterns were compared across four groups based on the results of the follow-up assessment: those who received an ASD diagnosis (n = 12), those at high risk for ASD and atypical development but with no ASD diagnosis (n = 22) and children with typical development at either low risk (n = 35) or high risk for ASD (n = 15; in the current review, the term ‘high risk’ refers to having high risk for ASD because of having one or more siblings with ASD). At six months, the groups did not differ in terms of their verbal or non-verbal developmental level, and non-verbal level at six months was used as a covariate in subsequent group comparisons. In contrast to their earlier toddler study described above [31], no interaction effect with condition was found. Rather, irrespective of condition, the ASD group looked less at the scene overall, less at the actress, and less at the face of the actress than the other groups (for the face, the comparison between the ASD and the low-risk group was only marginally significant). The groups did not differ with regard to looking time at specific face areas or at the toys surrounding the actress. A similar pattern was observed in another recent eye tracking study by the same group, which also suggested that compared to all other groups, 6-month-olds who later received an ASD diagnosis looked less at the inner features of the face when the face was speaking [35].

Also using a longitudinal design, Young *et al*. [36] studied looking performance in 6-month-olds at either low or high risk for ASD to see whether it was related to outcome 1.5 years later. Young *et al*. showed each infant presentations of his or her mother’s face on a TV monitor for three minutes, with bi-directional online visual and auditory feedback (that is, the child and the mother could influence each other’s behavior as in real life). Reconstructed fixation patterns, overlaid on the video stimulus, were mapped to areas of interest (AOIs) manually by trained coders. When children were two years old, they were classified into four groups: no concerns (n = 34), other concerns (n = 7), speech–language delay (n = 5), and ASD (n = 3). An experienced clinical psychologist made the classification based on the ADOS and other supplementary data. All categories except the speech–language delay category included both children at high risk and low risk for ASD. Because only three of the children in the study eventually received an ASD diagnosis, formal group comparisons were not possible. The authors found no indication that the infants later diagnosed with ASD looked at their mother’s face in an atypical way. Two of the children with ASD had a looking pattern at six months characterized primarily by eye fixations. In addition, regardless of risk status, fixating on the mouth of the mother at six months was associated with higher rates of language growth and larger vocabulary at two years. A similar finding was recently reported in a longitudinal study of high-risk and low-risk infants by Elsabbagh *et al*. [37].

Although covered by another recent review [38], we also briefly summarize studies focusing on how children with ASD look at faces shown in isolation [8,39-42]. Two studies (both using static stimuli) suggested that young children with ASD look less at key face areas and less at the mouth than typically developing children [39,41]. One of these reports indicated that looking patterns during face observation become more scattered in ASD during the early preschool years [39]. Another study found that looking patterns were more independent of face orientation (upright/inverted) in ASD than in typical children, in line with the view that children with ASD may process faces in a more piecemeal fashion [42]. Finally, as noted above, a recent study by Shic *et al*. [35] that included dynamic speaking faces found altered looking patterns to the inner part of faces already at six months in children later diagnosed with ASD. Importantly, the available eye tracking studies also indicate that the way children with ASD look at faces in such contexts relates both to the memory for the faces they observe [39] and to behavioral profiles (for example, language function) in everyday life [37,40].

### Discussion

It has been suggested that the earliest signs of autism could be found in the brain rather than at the behavioral level [43]. Thus, a central contribution of Chawarska *et al*.’s infant study [34] is the demonstration that behavioral signs of later diagnosed ASD are present as early as six months of age, which is equivalent to the earliest age that brain-based markers have been identified [8,13]. As the authors noted, decreased looking time at social aspects of the scene was not reflected in an increased looking time at objects. This finding speaks against the possibility that lack of looking time towards social stimuli arises from non-social objects being more salient or interesting for children with ASD. Interestingly, when these authors used the same stimuli with older children [31], they identified reduced attention to the face together with increased looking time towards non-social objects, which is in line with other studies of toddlers [44]. This outcome may indicate that increased attention to non-social objects is not the cause of low preference for social information but could be a consequence of it. However, another recent eye tracking study of preschoolers found that preference for faces presented together with objects was similar in children with ASD and in children with typical development except when the object belonged to categories such as trains, vehicles and airplanes [45]. When the object did belong to such categories, the children with ASD preferred to look less at the face compared to the other group. Against this background, the authors concluded that social attention in the preschool-aged children with ASD could be modulated by the salience of competing non-social objects. Manipulating the nature of the non-social object may be informative in future studies of even younger children.

One interpretation of the findings in the Chawarska *et al*. [31] toddler study is that the magnitude of group differences in looking performance was modulated by the degree of eye contact and other communicative signals included in the stimuli. In agreement with this view, an event-related potential (ERP) study by Elsabbagh *et al*. [8] found that gaze versus no-gaze manipulations (model looking towards or away from the child) had a differential effect on ERPs in 6- to 10-month-old infants, and that their later diagnostic status modulated the size of this effect. Exactly why people with ASD may react differently to eye contact is currently debated. First, it has been argued that altered eye contact effects in ASD can be explained by different patterns of arousal [46] or approach–avoidance tendencies [47]. Second, it has been suggested that individuals with ASD are impaired in fast subcortical processing of information from other people’s eyes [48]. An impairment in this system in ASD could be expected to cause different modulation of the social brain and associated sensory processing [49]. Interestingly, a recent eye tracking study by Elsabbagh *et al*. [50] found a normal ‘face pop out’ effect in infants later diagnosed with ASD. This finding speaks directly against the view that early altered behavioral and brain responses in ASD are primarily related to sub-cortical systems mediating an early face orienting bias.

A third possibility is that individuals with ASD fail to understand signals that indicate that someone else is intending to communicate with them. Such signals, which include direct gaze and infant-directed speech, are referred to as ostensive cues in the literature [51,52]. Ostensive cues enhance processing of subsequent referential communicative signals in infants with typical development [18]. It has also been emphasized that ‘children expect to learn something generalizable in ostensive–referential contexts rather than just become informed about particular episodic facts that obtain only in the ‘here-and-now” [51] (p. 151). Thus, in typical development, the presence of ostensive cues would be expected to facilitate learning of kind-specific entities (for example, object identity) while more transient aspects (for example, object position) would tend to be ignored in the presence of such cues. This hypothesis yields some testable questions regarding the mechanisms underlying socio-communicative impairments in ASD as well as their possible links to repetitive behaviors and lack of generalization. Eye tracking may be important for some of this testing. For example, based on the idea that contingent responding functions as an ostensive cue, one study used gaze-contingent eye tracking to show that a non-social object that ‘responded’ when being looked at can induce orientation following to specific targets in typically developing infants [52].

Worth noting, Chawarska *et al*. [34] observed no effect of context in their longitudinal infant sibling study with the same stimuli, which could suggest that the altered (lack of) modulation by context type seen in ASD emerges between infancy and toddlerhood. Also, two other eye tracking studies [32,53] demonstrated group differences in the complete absence of direct gaze (directed at the observing child).

Many of the reviewed studies have focused on how children with ASD look at faces. Neural correlates of face processing include both cortical and subcortical areas, and a large literature exists on the typical development of face processing and face perception [54]. In the context of dynamic faces accompanied by sound, the superior temporal sulcus is highly implicated, in addition to other core face areas such as the inferior occipital gyrus and the lateral fusiform gyrus [55]. Studies have indicated atypical face processing in children with ASD, for example, [56].

Several of the studies support the view that young infants and children with ASD look less at people and faces than typically developing children. However, none of the studies suggested that young infants (<12 months of age) who later receive an ASD diagnosis distribute their gaze across specific facial features (for example, mouth versus eyes) differently from control children [8,34-37]. In contrast, all the included toddler studies that examined how infants looked at the different features within a face found striking group differences [28,31,39]. However, these studies do not present a unified picture. The study by Jones *et al*. [28] found that two-year-olds with ASD looked less at the eyes and more at the mouth compared to control groups. In contrast, Chawarska and colleagues [31,39] studied a total of 68 one- to two-year-olds with ASD and found typical levels of looking time to the eyes and reduced looking time to the face and the mouth in this group. Among the studies of older preschool-aged children, some have reported that children with ASD tend to look less at the mouth than controls [41,57], but others have reported no group differences with regard to looking time to the eyes or mouth [42].

As described above, many methodological differences between the studies could potentially account for these divergent findings. For example, within the toddler studies, Jones *et al*. [28] included videos showing familiar child games while the Chawarska *et al*. study [31] included scenes that were probably rather unfamiliar to the infants. The Chawarska *et al*. [39] study included static faces. These discrepancies should motivate more systematic approaches in the future, manipulating key aspects of the stimuli [31,37]. It is difficult to compare the results from two studies using highly complex dynamic videos that were not related in a systematic way. Of note is that many studies have found that typically developing infants and children spend much time looking at the mouth, which could reflect that typical children use (audio-) visual information from the mouth to comprehend speech sounds [36,58-60].

Perhaps one of the clearest advantages with eye tracking over other methods is its ability to capture the dynamics of gaze behavior, even in complex environments [30]. In a social interaction, correct timing of gaze is likely to be critical, and a slight delay may mean missing important information, which will reduce the observer’s chances to engage in meaningful interactions with other people [53]. Later on, we discuss designs that study timing of gaze in rather constrained contexts. However, eye tracking is probably most useful in contexts that require both high spatial and high temporal resolution. Dynamics of gaze in naturalistic situations is probably the best example of such a context.

Against this background, it is noteworthy that many of the studies reviewed above focused on the spatial aspects of looking patterns, essentially asking the question of ‘where do the different groups look?’ This approach is common in eye tracking research more generally. With it, researchers usually define one or several AOIs, calculate aggregated looking time scores within these areas, and use these values as dependent measures in their analyses. The reviewed studies underscore the relevance of this approach. At the same time, the classical AOI method does not exploit the full potential of the eye tracking technique. Holmqvist and colleagues [25] provide a guide to different analytic approaches for tracking data (see also [20,30,61-67]). We recently illustrated how bottom-up analytic methods can be used to identify and visualize group differences in complex eye movement data recorded during observation of semi-naturalistic scenes [53], as shown in Figure 1a (see also [68]).

When comparing groups in terms of their spatial looking patterns, high-quality calibration and re-calibration are essential. Moreover, the calibration stimuli should be chosen with care to ensure that the groups look at these in a similar manner. It is advantageous to measure and report the actual spatial accuracy of the data (separately for each group), rather than to rely on the figures reported by the eye tracker manufacturer. The actual spatial resolution will have important consequences for the definition of AOIs and for subsequent group comparisons.

Future studies of naturalistic viewing can advance in many directions. First, one can systematically evaluate results across different types of scenes. This approach, employed by some of the reviewed studies [31], potentially both decreases the number of possible interpretations and increases the ecological validity of the study. Second, live eye tracking technology [69,70] could be used to enhance ecological validity further. In a recent study by Noris *et al*. [69], the authors collected live eye tracking data from children with ASD in a naturalistic setting, using a head camera that recorded both the scene and a close-up of the child’s eyes (via a small mirror located above the eyes). In addition to the obvious ecological advantage, live presentation can also be important for the internal validity of the study. For example, a study of adults showed that different skin conductance patterns in response to eyes that were either open or closed were present only when the stimuli were presented live [71]. Although live eye tracking has its own difficulties (for example, variability in presentations across participants), it would be ideal for addressing many questions related to ASD given that problems with social interaction are characteristic of this disorder.

Third, control experiments/tasks could be embedded in the eye tracking battery to relate complex viewing patterns to eye tracking measures with more established neural, oculomotor, or cognitive correlates. For example, this approach could involve relating naturalistic viewing to disengagement latencies measured in a dedicated experimental task (see below for examples). Fourth, investigations could address whether the gaze location (or other eye tracking measures) can be explained by specific stimulus properties that are objectively quantifiable in space and/or time. Such properties include luminance, contrast, motion, audiovisual synchrony and even social aspects [29,53,72]. The causal link between such properties and looking is strengthened with exploitation of the time-series nature of eye tracking data to demonstrate close time dependencies. Finally, naturalistic scenes could be used as a way to sample basic and ecologically valid eye movement data and compare these across groups and across contexts.

### Studies using the paired visual preference paradigm

In the paired preference paradigm, two visual displays that differ along one or more dimensions are presented side-by-side on a screen. This type of stimuli has a long tradition in developmental psychology [73]. Frequently, the logic behind this approach is to be able to link looking time to a specific type of information. Thus, the fewer stimulus dimensions along which the two sides differ, the easier it will be to interpret the results. If processing of the information in question has established brain correlates, the results also will have implications at a neural level. Manipulating only one dimension at a time is difficult to accomplish, and follow-up experiments are therefore often needed to exclude alternative explanations [74].

Two eye tracking studies have used the paired visual preference paradigm to study preference for biological motion and audiovisual synchrony in ASD [29,74]. Klin *et al*. [29] applied the paradigm to study the influence of biological motion and audiovisual synchrony on looking patterns in children with ASD (subsample 1, n = 21, mean age 2.2 years; subsample 2, n = 10, mean age = 2.1 years), children with typical development (n = 39, mean age 2.0 years) and children with developmental delays (n = 16, mean age = 2.0 years). The ASD group was matched to the other two groups in terms of non-verbal ability, and to the developmentally delayed group in terms of verbal ability. The stimuli were several different movies showing point light animation pairs accompanied by sound (30 seconds each; ASD subsample 2 was shown two other movies of the same length, and this group was included as a validation sample). One animation was presented upright and played forward; the other was shown upside-down and played in reverse. The authors tested each group’s preference for the upright animation (indicative of preference for biological motion) and whether the groups oriented to audiovisual synchrony. Results suggested an absence of preference for biological motion in ASD, combined with a tendency to orient towards spatial locations with much audiovisual synchrony, such as synchrony produced by clapping hands. The control groups oriented to biological motion with no indication that these groups oriented to audiovisual synchrony. This study strongly suggests that reduced (or, even, the complete absence of) preference for biological motion may be characteristic of very young children with ASD. Given the putative role of biological motion in typical development [75,76], this finding may have strong theoretical implications for our understanding of the altered developmental trajectories in ASD.

In addition, the study by Klin *et al*. presented a strong and testable hypothesis regarding the role of audiovisual synchrony for visual orienting in ASD. This hypothesis was the focus of a study by Falck-Ytter *et al*. [74], who created a point light display similar to the one used by Klin *et al*. However, rather than comparing visually dissimilar point light animations, the visual information was kept constant across conditions that differed only in the spatial distribution of audiovisual synchrony. The auditory signals (clapping) were manipulated so that they either occurred in synchrony with the upright animation or the inverted animation of the pair (recorded from a person standing up, clapping hands). The study included a group with ASD (autistic disorder only, n = 10, age 3.4 years), a group with typically developing 3-year-olds (n = 14, age 3.5 years), as well as a group of typically developing toddlers (n = 11, 1.4 years). In contrast to the Klin *et al*. study, the results showed that the typically developing groups were strongly influenced by audiovisual synchrony while the ASD group was not. A follow-up experiment confirmed that the groups differed in terms of their preference for biological motion, with the ASD group performing at chance level.

Pierce *et al*. [44] used the paired visual preference paradigm to ask whether toddlers with ASD prefer to look at dynamic geometric images rather than dynamic social images, and whether looking pattern in this context can be used to classify a toddler as having ASD. The study included a group with ASD (n = 37, mean age 2.3 years), typical development (n = 51, mean age 2.1 years) and developmental delay (n = 22, mean age = 1.9 years). The ASD and the developmentally delayed group were matched on verbal, non-verbal and adaptive-functional levels. The stimulus was a one-minute movie showing a computer screen saver animation on one side and children in high action (for example, doing yoga) on the other. Results showed that the children with ASD looked for a shorter time at the social side (relative to the non-social side) than the two other groups. The proportion of children with ASD showing preference for the screen saver side was greater than the corresponding proportion in the two control groups. One of the most interesting suggestions from this study was that there seemed to be two subgroups of children with ASD, some preferring the non-social screen saver and some preferring the social videos, and that these groups could be distinguished on the basis of a one-minute eye tracking session. Although the authors did not formally explore the idea, the data visually presented in the article strongly suggest different distributions of preference scores across the three groups of children (bimodal in ASD, normal in the two control groups).

### Discussion

In all preferential looking studies reviewed in this section, one side of the screen included more social information than the other. All studies found a clear preference in non-autistic toddlers for the ‘social side’ while the children with ASD showed no such preference. This pattern evokes a general question of whether the differences should be seen as reflecting differences in information processing or in motivation. One could interpret the results as support for the social motivation theory of ASD [77,78], implying that the lack of preferential looking reflects lack of reward associated with looking at the social scene (or heightened reward associated with looking at the non-social scene). Alternatively, or in addition, the differences can be interpreted as reflecting reduced detection of, or sensitivity to, certain information in ASD. This ambiguity also applies to many of the results from the (semi-) naturalistic scenes reviewed earlier, and is hard to resolve solely on the basis of aggregated looking time measures.

The studies by Klin *et al*. and Falck-Ytter *et al*. differ in their conclusions regarding audiovisual synchrony. Klin *et al*. found that children with ASD orient to this type of information more strongly than other children when it is embedded in point light displays of biological motion, but Falck-Ytter *et al*. found the opposite effect. Each study had its own strengths and weaknesses. Klin *et al*. included relatively large samples and two control groups that were better matched to the ASD group on verbal and non-verbal function than the Falck-Ytter *et al*. groups. Falck-Ytter *et al*., on the other hand, included a selective manipulation of audiovisual synchrony. The complex picture arising from these studies should motivate investigations that include well-matched samples combined with designs that allow unambiguous assessment of audiovisual synchrony preference.

The Pierce *et al*. study [44] may serve as an illustration of a specific kind of methodological challenge associated with eye tracking. Different streams of data from an eye tracking session (such as gaze location on the screen, pupil size, blinks, fixation duration and so on) can be independent of each other, yet are sometimes either directly or indirectly related, and these instances should be identified. Thus, although one should try to exploit the possibilities of the eye tracking method, one also should pay attention to potential ‘within-eye-tracker confounds’. Pierce *et al*. [44] reported that children with ASD tended to look more towards the screen saver side than non-ASD children. In addition, children with ASD who preferred to look at screen savers had a lower fixation rate when looking at these non-social stimuli compared to all other child groups. Long fixations have been interpreted as an index of increased stimulus processing, and on the basis of these results, Pierce *et al*. concluded that, ‘While a preference for geometric patterns alone may be an intriguing novel identifier of early autism, results also illustrated a distinct pattern of saccades within the geometric responders [children preferring to look at the screensaver]’ and that ‘the combination of a preference for geometry combined with saccade quantity might be a particularly strong early identifier of autism’ (pp. 107–108). However, this suggestion remains speculative given that no data were presented to support the view that the looking time measure and the saccade frequency measure (fixation rate) reflected independent processes. Alternatively, interest in a particular type of object increases the fixation length on that object (and thus decreases the fixation rate), leading to more aggregated looking time at that object as well.

In the context of this review, it is worth noting that the paired visual preference paradigm does not require eye tracking technology. For example, a non–eye tracking study by Tek *et al*. [15] used the preferential looking paradigm to study the mechanisms by which young children with ASD and typical development learn novel words. Finally, as with the naturalistic scenes, it may be important to complement studies of preferential looking with assessments of oculomotor and general attentional functions (see below). A general attentional dysfunction could significantly affect performance in both of these paradigms.

### Event-related designs

Event-related designs typically focus on properties of gaze shifts (for example, latency, accuracy), and the paradigms included in this section have a more experimental flavor than the (semi-) naturalistic approaches.

Using an event-related paradigm called the gap overlap task, Elison *et al*. [17] investigated saccade latencies in infants at high and low risk for ASD followed from seven to 25 months of age and correlated these measures to structural brain measures. In each trial, the latencies of gaze shifts from a central cue to a peripheral cue were measured. During gap trials, the central cue disappeared prior to the onset of the peripheral cue. In the overlap conditions, the central stimulus remained on screen throughout the entire trial. Saccadic latencies are typically larger in this condition because the observer has to both disengage from the central cue and orient to the peripheral cue. Performance in the overlap condition was considered to be a measure of visual orienting while performance in the gap condition was considered to be a measure of oculomotor efficiency. Both eye tracking and magnetic resonance imaging were conducted at seven months. Risk status and ADOS scores at follow up were used to assign the children to either the low-risk control group (n = 41), high-risk–positive group (n = 16), or high-risk–negative group (n = 40). The groups did not differ in terms of verbal or non-verbal level of functioning at the six-month visit. Results showed that performance in the gap condition was lower in the high-risk–positive group than in the low-risk controls (the high-risk–negative group did not differ from either of the other two groups). In the low-risk group only, performance on this task was related to the radial diffusivity in the left corticospinal tract. In terms of performance in the overlap condition, latencies in the high-risk–positive group were significantly longer than in both of the other groups. In the low-risk controls only, performance in this condition was related to radial diffusivity in the splenium of the corpus callosum. In addition to providing another example of subtle early behavioral cues predicting the severity of symptoms years later, this study suggests a specific neural candidate for this difference in visual orienting. Given that previous research has documented widespread white matter tract alterations between high-risk–positive and high-risk–negative children, a fascinating prospect for further study is the possibility of relating other neural pathways to distinct behavioral functions in ASD.

While Elison studied saccadic reaction times, Falck-Ytter [79] used eye tracking to study predictive eye movements in ASD. The study was motivated by the fact that predictive eye movements during action observation are linked to perception–action circuits in the brain [80], circuits that have been proposed to be dysfunctional in ASD [56,81]. Movies showing manual actions were played for the children, and the arrival of the children’s gaze to the action goals was related to the arrival of the moving hand in the movies. Typically in such designs, if the gaze arrives before the hand does, the gaze shift is considered predictive [82]. The study included 18 children with ASD (mean age 5.1) and 13 typically developing controls (mean age 5.0 years) as well as an adult group. Results showed that all groups used predictive eye movements in action observation; thus, the findings provided no support for the view that there is a fundamental action prediction problem in ASD [83]. Interestinly, another study reported that in a more complex task involving two people engaged in a conversation, children with ASD did not follow the turn-taking of the conversation in a predictive manner [68].

Yet another event-related paradigm of particular relevance for autism research is the gaze following task. Bedford *et al*. [84] used a similar longitudinal design as the previously described studies by Chawarska *et al*. [34] and Young *et al*. [85] to map gaze following performance in infants in relation to later diagnosis. Stimuli were based on a previous study [86] and showed a female model looking into the camera and then turning her gaze to one of two objects placed on a table in front of her. Gaze following was operationalized as gaze shifts going from the model’s face to the correct object. The group that later received an ASD diagnosis (n = 12) was compared to infants with low risk (n = 38) as well as to high-risk infants with either typical (n = 14) or atypical (n = 9) development at follow-up. Eye tracking was conducted at seven and 13 months of age. Diagnostic categorization was made using information from multiple visits by members of the research team. The low-risk controls differed from all other groups with regards to their developmental level at seven months, and explicit language level (at either seven or 13 months) was used as a covariate in the main analysis. Results showed no differences on any eye tracking measure at seven months. At 13 months, the groups did not differ in terms of gaze following accuracy (that is, shifting gaze to the attended rather than the unattended object). However, both the ASD group and the non-autistic group with developmental delays showed less preferential looking to the attended object. The authors also reported that the latter measure was negatively related to autistic symptomatology in the children with high risk for ASD, as measured with the ADOS. This study is in line with other studies showing that automatic gaze cueing is intact in young children with ASD [87], but indicates that children with elevated levels of socio-communicative impairments (measured with the ADOS) have a tendency to show less sustained looking towards the attended object. The correlation between autistic symptoms and looking time was found while controlling for language level and is in line with Navab *et al*. [88], who reported that in a large sample of 18-month-olds at high risk for ASD, there was a marginally significant negative association between sustained looking towards the attended object and socio-affective symptomatology measured with the ADOS. Interestingly, a study by Gliga *et al*. [16] found that in three-year-olds at risk for ASD, the level of socio-communicative impairments modulated both the looking time to the attended object and the tendency to learn the name of that object. Moreover, Bedford *et al*. [89] suggested that failure to establish stable object–word mappings could be related to low sensitivity to feedback cues during the learning process in ASD. Finally, gaze following accuracy in this paradigm seems to be related to adaptive communication in children with autistic disorder as old as six years [90].

### Discussion

The studies reviewed in this section illustrate the value of event-related eye tracking measures for understanding aspects of oculomotor performance, visual orienting, action prediction and gaze following.

The results by Elison *et al*. point to the possibility that basic attentional functions are already impaired in ASD during the first year of life. This conclusion is also supported by Elsabbagh *et al*. (see also [91,92]), although that study suggested that problems with visual orienting develop somewhat later. Together, these data introduce a challenge for eye tracking studies focusing on other early functions that may be affected by oculomotor and visual orienting ability.

The studies by Elison *et al*. [17] and Elsabbagh *et al*. [91,92] had some notable differences. In contrast to Elison *et al*., who used static cues, Elsabbagh *et al*. used a dynamic central cue (that became static simultaneously with the onset of the peripheral cue in the overlap condition). Another important difference was that Elsabbagh *et al*. [91,92] included a baseline condition to which both the gap and the overlap conditions were compared while Elison *et al*. [17] based their analyses on the latency scores from the two conditions directly [17]. Furthermore, while Elison *et al*. used corneal reflection eye tracking, Elsabbagh *et al*. extracted latencies from video recordings of eye movements. Finally, Elsabbagh *et al*. used non-social stimuli, while Elison used mixed social and non-social stimuli. Interestingly, in a study of toddlers, Chawarska *et al*. [93] found no differences for non-social stimuli, but that ASD children were faster disengaging from social stimuli (neutral faces looking with either direct or averted gaze) than control groups.

In light of these findings and because studies of ASD are orienting toward younger populations, it may be useful to briefly review some basic findings related to the typically developing oculomotor system (for a review of oculomotor function in older children with ASD, see Simmons *et al*. [94]). At birth, infants can direct their gaze to interesting sights in the environment, primarily using saccadic eye movements [95]. These rapid fixation shifts from one location to another are variable in newborns and often involve several hypometric saccades that successively bring an object of interest closer to the infant’s focal point [96]. As infants grow older, saccade latencies decrease and fewer corrective saccades are needed before the target is fixated [97,98]. Around two months of age, infants also gain the ability to track moving objects with smooth eye movements, called ‘smooth pursuit’ [99]. This tracking is initially reactive, and saccades are frequently used to reposition the eyes on the moving target. By four months of age, infants track objects moving in the horizontal plane in a smooth manner [100]. The hallmark of this development is the ability to track external events with predictive eye movements; that is, the ability to keep up with external events without a lag. The challenge is to overcome the internal processing lag of the oculomotor system and plan eye movements with respect to future events. In this regard, eye movements are no different from other actions that are organized around future states and goals [101]. Evidence of predictive tracking is visible by two- to four-months of age, although the oculomotor system continues to fine-tune over the first year of life. For example, vertical and two-dimensional tracking matures slowly over the first years [102,103], and saccade latencies decrease continuously during infancy [98] and childhood [104]. Saccade latency is highly variable among individuals at these ages. Finally, in the context of autism research, it may be important to note that a number of factors, including arousal, influence saccade parameters [105]. Thus, even measures as ‘simple’ as saccadic reaction times may be rather complicated to interpret and need to be put into a developmental context.

The study by Elison *et al*. also illustrates how eye tracking and brain-based measures can be linked, which clearly is a priority for future research. Although plausible neural mechanisms for some eye tracking measures have been established [80] (see also [106,107]), this is not the case in general, and certainly not when it comes to human infants. One study suggested that spontaneous gazing towards the eye area is linked to increased amygdala activity in older children with ASD [46]. Key and Stone [108] reported that event-related potentials in infants in response to changes in facial features were associated with producing fewer fixations on the irrelevant aspects of the stimuli. Elison *et al*. [17] suggested that in neurotypical human infants, visual orienting and oculomotor efficiency are uniquely related to microstructural organization of the splenium of the corpus callosum and the left corticospinal tract, respectively, and cited animal research linking oculomotor efficiency with activity in the superior colliculus [109]. The ‘face pop out’ effect, which was recently studied with eye tracking in infants at risk for ASD, has been associated with fast and automatic subcortical face processing [50]. Processing of biological motion, which can be studied using the preferential looking paradigm [29,110,111], has been linked to activity in the superior temporal sulcus [112]. Finally, results from (semi-) naturalistic viewing paradigms with social content are often linked to processing in the ‘social brain network’ [31], but the exact nature of these associations is largely unknown; for two related reviews, see [77,113].

## Conclusions

This review has covered eye tracking studies of early autism, ranging from research that involved viewing of naturalistic scenes to highly experimental designs. We have argued that future research can benefit from taking more advantage of the unique options provided by eye tracking [25,53,57]. Such analyses ask both where the participant looks, but also – and equally important – when the participant looked there. Gaze, both in non-social and social contexts, is a highly dynamic phenomenon, and to capture it requires better use of both the spatial and the temporal resolution of the eye tracker. Integration is needed of explorative naturalistic approaches with experimental paradigms and measures, as well as with more advanced analytic approaches that can constrain interpretations.

What are the substantial findings from this body of research? Several of the reviewed studies have found that reduced looking time to people and faces is characteristic of young infants and toddlers with ASD [31,32,34,35]. In toddlers with ASD, altered looking patterns across facial parts such as the eyes and mouth have been found [28,39], as has a failure to orient to biological motion [29,74]. Of note, early group differences are not restricted to purely social tasks. One of the identified studies suggested that visual orienting is altered in young infants who have high levels of autistic symptomatology at age two years [17] (see also [91]). The same study provided data suggesting links between specific eye tracking measures and specific brain structures. Reduced capacity for disengaging attention could have negative effects both on arousal regulation and joint attention behaviors, such as gaze following [114].

This review also covers some controversies. One concerns how young children with ASD look at faces, in particular their looking time to other people’s eyes and mouths. The reviewed studies indicate that looking time to eyes and mouth probably depends on a number of contextual and participant factors (diagnostic status being only one of many) that are currently relatively poorly understood. However, it now seems fair to conclude that looking time to the mouth is related to language function at specific early periods in typical development [36,58]. Another controversy has to do with the degree to which typical children and children with ASD tend to orient to audiovisual synchrony embedded in point light displays of biological motion [29,74]. It has been suggested that children with ASD look to the mouth because they tend to orient to audiovisual synchrony [29], so the ‘facial feature’ and ‘audiovisual synchrony’ controversies are in fact related.

For researchers not familiar with eye tracking, it can be difficult to realize the diversity of the questions the method can address. In fact, a full overview of the possibilities associated with it is outside the scope of this article [25]. The span may be broader than for most other available methods. It is possible to ask questions related to autonomic responses [115] and other neural functions [17,82], as well as specific (oculo-)motor [98], attentional [17], perceptual [116], cognitive [18], and emotional [117] processes. In addition, somewhat similar to conventional personality tests, eye tracking can be used to investigate spontaneous visual preferences and interests in complex situations that resemble real life [27]. Although some eye tracking measures are obtainable with different (often more time-consuming and less exact) means [44], several other highly meaningful measures are simply not accessible without recordings of the movements of the eyes with very high spatiotemporal resolution [118]. The method is non-invasive and does not require more than an ability to move the eyes, making it ideal for the study of young children and infants. Furthermore, because many eye tracking measures are intuitively meaningful, data from such research is often well received by a broader audience (for example, showing dynamic gaze patterns superimposed on the stimuli). In this respect, eye tracking can provide an important way of communicating scientific results to non-specialists, including parents and other stakeholders. It is conceivable that eye tracking can be used as an integrated part of screening and diagnostic assessments (and potentially even training) in the future. Some examples of eye tracking tasks are given that discriminate reasonably well between children with ASD and non-autistic children at an individual level [44,53]. Also, some studies have used eye tracking to characterize sub groups with autism [119,120], which may be useful for understanding the heterogeneity of the disorder. However, although these results are promising, the clinical value of eye tracking remains to be established.

In sum, although eye tracking has some drawbacks (primarily high cost and expertise requirements), there is a great potential to exploit and develop this method further in the field of early autism. Eye tracking data can be conceptualized as describing autism at a unique, intermediate level, with links ‘down’ to underlying neurocognitive networks, as well as ‘up’ to everyday function and dysfunction. By describing these links in detail, eye tracking will reveal important features of the complex picture of autism.

## Consent

Written informed consent was obtained from the children's guardian/parent/next of kin for the publication of this report and any accompanying images.

## Abbreviations

ADOS: Autism diagnostic observation schedule; AOI: Area of interest; ASD: Aautism spectrum disorder; ERP: Event-related potential; SLL: Specific language disorder.

## Competing interests

The authors declare that they have no competing interests.

## Authors’ contributions

TFY wrote the paper with contributions from SB and GG. All authors read and approved the final manuscript.

## Supplementary Material

Additional file 1 List of identified eye tracking studies. Click here for file
