# Supplementary material for: Eye tracking in early autism research
Source: J Neurodev Disord. 2013 Sep 26;5(1):28. doi: 10.1186/1866-1955-5-28 (PMC3849191; doi:10.1186/1866-1955-5-28)
Supplement: Additional file 1 — List of identified eye tracking studies. [file 1866-1955-5-28-S1.docx]

**Appendix A: List of identified eye tracking studies**

**Studies of infants and toddlers**

Bedford, R., Elsabbagh, M., Gliga, T., Pickles, A., Senju, A., Charman, T., . . . Team, B. (2012). Precursors to Social and Communication Difficulties in Infants At-Risk for Autism: Gaze Following and Attentional Engagement. Journal Of Autism And Developmental Disorders, 42(10), 2208-2218. doi: 10.1007/s10803-012-1450-y

Bedford, R., Gliga, T., Frame, K., Kudry, K., Chandler, S., Johnson, M. H., & Charman, T. (2013). Failure to learn from feedback underlies word learning difficulties in toddlers at risk for autism. Journal of Child Language, 40, 29-46.

Campbell, D., Shic, F., Macari, S., Chawarska, K (in press). Gaze Response to Dyadic Bids at 2 Years Related to Outcomes at 3 Years in Autism Spectrum Disorders: A Subtyping Analysis. Journal Of Autism And Developmental Disorders.

Chawarska, K., Macari, S., & Shic, F. (2012). Context modulates attention to social scenes in toddlers with autism. Journal Of Child Psychology And Psychiatry, 53(8), 903-913.

Chawarska, K., Macari, S., & Shic, F. (in press). Decreased Spontaneous Attention to Social Scenes in 6-Month-Old Infants Later Diagnosed with Autism Spectrum Disorders. Biological Psychiatry. doi: dx.doi.org/10.1016/j.biopsych.2012.11.022

Chawarska, K., & Shic, F. (2009). Looking But Not Seeing: Atypical Visual Scanning and Recognition of Faces in 2 and 4-Year-Old Children with Autism Spectrum Disorder. Journal Of Autism And Developmental Disorders, 39(12), 1663-1672. doi: 10.1007/s10803-009-0803-7

Elison, J. T., Paterson, S. J., Wolff, J. J., Reznick, J. S., Sasson, N. J., Gu, H., . . . Piven, J. (in press). White Matter Microstructure and Atypical Visual Orienting in 7-Month-olds at Risk for Autism. American Journal Of Psychiatry.

Elsabbagh, M., Bedford, R., Senju, A., Charman, T., Pickels, A., & Johnson, M. (in press). What you see is what you get: Contextual modulation of face scanning in typical and atypical development. Social Cognitive And Affective Neuroscience.

Elsabbagh M, Gliga T, Pickels A, Hudry K, Charman T, Johnson M, the BASIS team (in press). The development of face orienting in infants at-risk for autism. Behavioural Brain Research.

Elsabbagh, M., Mercure, E., Hudry, K., Chandler, S., Pasco, G., Charman, T., . . . the BASIS team. (2012). Infant Neural Sensitivity to Dynamic Eye Gaze Is Associated with Later Emerging Autism. Current Biology, 22(4), 338-342. doi: 10.1016/j.cub.2011.12.056

Jones, W., Carr, K., & Klin, A. (2008). Absence of preferential looking to the eyes of approaching adults predicts level of social disability in 2-year-old toddlers with autism spectrum disorder. Archives Of General Psychiatry, 65(8), 946.

Key, A. P. F., & Stone, W. L. (2012). Same but Different: 9-Month-Old Infants at Average and High Risk for Autism Look at the Same Facial Features but Process Them Using Different Brain Mechanisms. Autism Research, 5(4), 253-266. doi: 10.1002/aur.1231

Klin, A., Lin, D. J., Gorrindo, P., Ramsay, G., & Jones, W. (2009). Two-year-olds with autism orient to non-social contingencies rather than biological motion. Nature, 459(7244), 257-261.

Merin, N., Young, G. S., Ozonoff, S., & Rogers, S. J. (2007). Visual fixation patterns during reciprocal social interaction distinguish a subgroup of 6-month-old infants at-risk for autism from comparison infants. Journal Of Autism And Developmental Disorders, 37(1), 108.

Navab, A., Gillespie-Lynch, K., Johnson, S. P., Sigman, M., & Hutman, T. (2012). Eye-Tracking as a Measure of Responsiveness to Joint Attention in Infants at Risk for Autism. Infancy, 17(4), 416-431. doi: 10.1111/j.1532-7078.2011.00082.x

Pierce, K., Conant, D., Hazin, R., Stoner, R., & Desmond, J. (2011). Preference for Geometric Patterns Early in Life As a Risk Factor for Autism. Archives Of General Psychiatry, 68, 101-109.

Shic, F., Bradshaw, J., Klin, A., Scassellati, B., & Chawarska, K. (2011). Limited activity monitoring in toddlers with autism spectrum disorder. Brain Research, 1380, 246-254. doi: 10.1016/j.brainres.2010.11.074

Shic, F., Macari, S., Chawarska, K. (in press). **Speech Disturbs Face Scanning in 6-Month-Old Infants Who Develop Autism Spectrum Disorder.** *Biological Psychiatry*.

Young, G. S., Merin, N., Rogers, S. J., & Ozonoff, S. (2009). Gaze behavior and affect at 6 months: predicting clinical outcomes and language development in typically developing infants and infants at risk for autism. Developmental Science, 12(5), 798-814.

**Studies of preschool-aged children**

de Wit, T., Falck-Ytter, T., & von Hofsten, C. (2008). Young children with Autism Spectrum Disorder look differently at positive versus negative emotional faces. Research In Autism Spectrum Disorders, 2(4), 651-659.

Falck-Ytter, T. (2008). Face Inversion Effects in Autism: A Combined Looking Time and Pupillometric Study. Autism Research, 1(5), 297-306.

Falck-Ytter, T. (2010). Young children with autism spectrum disorder use predictive eye movements in action observation. Biology Letters, 6(3), 375-378.

Falck-Ytter, T., Fernell, E., Gillberg, C., & von Hofsten, C. (2010). Face scanning distinguishes social from communication impairments in autism. Developmental Science, 13(6), 864-865.

Falck-Ytter, T., Fernell, E., Lundholm Hedvall, Å., von Hofsten, C., & Gillberg, C. (2012). Gaze performance in children with autism spectrum disorder when observing communicative actions. Journal Of Autism And Developmental Disorders, 42, 2236-2245. doi: DOI 10.1007/s10803-012-1471-6

Falck-Ytter, T., Rehnberg, E., & Bölte, S. (2013). Lack of Visual Orienting to Biological Motion and Audiovisual Synchrony in 3-Year-Olds with Autism. Plos One.

Falck-Ytter, T., von Hofsten, C., Gillberg, C., & Fernell, E. (in press). Visualization and analysis of eye movement data from children with typical and atypical development. Journal Of Autism And Developmental Disorders.

Gliga, T., Elsabbagh, M., Hudry, K., Charman, T., Johnson, M. H., & Team, B. (2012). Gaze Following, Gaze Reading, and Word Learning in Children at Risk for Autism. Child Development, 83(3), 926-938. doi: 10.1111/j.1467-8624.2012.01750.x

Hosozawa, M., Tanaka, K., Shimizu, T., Nakano, T., & Kitazawa, S. (2012). How Children With Specific Language Impairment View Social Situations: An Eye Tracking Study. Pediatrics, 129(6), E1453-E1460. doi: 10.1542/peds.2011-2278

Nakano, T., Tanaka, K., Endo, Y., Yamane, Y., Yamamoto, T., Nakano, Y., . . . Kitazawa, S. (2010). Atypical gaze patterns in children and adults with autism spectrum disorders dissociated from developmental changes in gaze behaviour. Proceedings Of The Royal Society B-Biological Sciences, 277(1696), 2935.

Noris, B., Nadel, J., Barker, M., Hadjikhani, N., & Billard, A. (2012). Investigating Gaze of Children with ASD in Naturalistic Settings. Plos One, 7(9), e44144. doi: doi:10.1371/journal.pone.0044144

Sasson, NJ., Touchstone, EW. (in press). Visual Attention to Competing Social and Object Images by Preschool Children with Autism Spectrum Disorder. *Journal Of Autism And Developmental Disorders.*

von Hofsten, C., Uhlig, H., Adell, M., & Kochukhova, O. (2009). How children with autism look at events. Research In Autism Spectrum Disorders, 3(2), 556-569.
